# Implementation fidelity of nutritional counselling, iron and folic acid supplementation guidelines and associated challenges in rural Uasin Gishu County Kenya

**DOI:** 10.1186/s40795-020-00403-1

**Published:** 2020-12-18

**Authors:** Roselyter Monchari Riang’a, Anne Kisaka Nangulu, Jacqueline E. W. Broerse

**Affiliations:** 1 Current address: Department of Sociology, Psychology and Anthropology, School of Arts and Social Sciences, Moi University, Eldoret, Kenya; 2 Current address: Principle, Bomet University College, Bomet, Kenya; 3 Current address: Department of History and political Science, School of Arts and Social Sciences, Moi University, Eldoret, Kenya; 4 Current address: Athena Institute, Faculty of Science, Vrije Universiteit Amsterdam, and Amsterdam Public Health Research Institute, Amsterdam, The Netherlands

**Keywords:** Maternal nutrition, Pregnancy, Programme evaluation, Implementation fidelity, Nutrition interventions, Nutrition education and counselling, Iron and folic acid supplementation, Kalenjin, Kenya

## Abstract

**Background:**

Implementation fidelity which is defined as the degree to which programmes are implemented as intended is one of the factors that affect programme outcome, thus requiring careful examination. This study aims to acquire insight into the degree to which nutritional counselling and Iron and Folic Acid supplementation (IFAs) policy guidelines during pregnancy have been implemented as intended and the challenges to implementation fidelity.

**Methods:**

Data were collected in rural Uasin Gishu County in the western part of Kenya through document analysis, questionnaires among intervention recipients (*n* = 188) and semi-structured interviews with programme implementers (*n* = 6). Data collection and analysis were guided by an implementation fidelity framework. We specifically evaluated adherence to intervention design (content, frequency, duration and coverage), exposure or dosage, quality of delivery and participant responsiveness.

**Results:**

Coverage of nutritional counselling and IFAs policy is widespread. However, partial provision was reported in all the intervention components. Only 10% accessed intervention within the first trimester as recommended by policy guidelines, only 28% reported receiving nutritional counselling, only 18 and 15% of the respondents received 90 or more iron and folic acid pills respectively during their entire pregnancy period, and 66% completed taking the IFAs pills that were issued to them. Late initial bookings to antenatal care, drug stock shortage, staff shortage and long queues, confusing dosage instructions, side effects of the pills and issuing of many pills at one go, were established to be the main challenges to effective implementation fidelity. Anticipated health consequences and emphasis by the health officer to comply with instructions were established to be motivations for adherence to nutritional counselling and IFAs guidelines.

**Conclusions:**

Implementation fidelity of nutritional counselling and IFAs policy in Kenya is generally weak. There is need for approaches to enhance early access to interventions, enhance stock availability, provide mitigation measures for the side effects, as well as intensify nutritional counselling to promote the consumption of micronutrient-rich food sources available in the local environment to substitute for the shortage of nutritional supplements and low compliance to IFAs.

## Background

Maternal malnutrition is a global problem and is more prevalent in low- and middle-income-countries (LMICs). In Kenya, many pregnant women have poor nutritional status, with 42% suffering from anaemia [1] and 12.3% of women of reproductive age having a BMI of less than 18.5 [2]. Low birth weight (< 2500 g), one of the best composite indicators of short- and long-term undernutrition in women affects one in ten new-borns in Kenya [2]. Anaemia in pregnancy contributes to high rates of intrauterine growth retardation (IGR) and premature birth, increased complications of post-partum bleeding and greater risk of maternal mortality [3–7].

Malnutrition is a complex problem which is caused by a wide range of direct and indirect factors including inadequate nutritional intake as a result of household food insecurity or an infection which can increase nutritional requirements and prevent the body from absorbing those consumed [8]. For women in sub-Saharan Africa, the environmental and economic conditions place an extra burden on their nutritional status. Pervasive poverty affects the quality of their diet, their heavy workload increases their nutritional requirements, frequent and short reproductive cycles often leave them moving from one pregnancy to the next without adequately replenishing the body’s nutrient stores, and lack of nutritional knowledge makes them consume inappropriate nutrition [9].

The Government of Kenya has acknowledged the problem of malnutrition and is committed to reducing hunger and to achieve adequate nutrition for the optimum health of all Kenyans as a fundamental human right [10]. Since 2001, maternal nutrition interventions in Kenya were implemented within the framework of the Kenya Reproductive Health strategy (1997–2010) using the World Health Organization (WHO) Focused Antenatal Care (FANC) strategy. FANC put in place a National Reproductive Health Programme that sought to expand on the achievements of the Maternal Child Health/Family Planning (MCH/FP) programme that had been functioning since 1967. The goal of the programme was to provide a comprehensive and integrated system of reproductive health care that offers a full range of services by the Government, Non-Governmental organizations (NGOs) and the Private Sector. In the FANC care package, nutrition education and counselling are the main strategy to improve the nutritional status of women during pregnancy.

In 2013, the Maternal Infant and Young Child Nutrition (MIYCN) policy for health workers was introduced [11]. This is anchored in WHO’s the Essential Nutrition Actions: Improving maternal, new-born, infant and young health and nutrition [12]. In the MIYCN Programme, nutrition education and counselling still remain the main strategy to improve the nutritional status of women during pregnancy. The strategy focuses primarily on promoting a healthy diet by increasing the diversity and amount of food consumed, promoting adequate weight gain through sufficient and balanced protein and energy intake as well as promoting consistent and continued use of micronutrient supplements (including IFAs), food supplements or fortified foods. IFAs is offered free of charge in all Kenyan government hospitals as part of routine ANC services.

The MIYCN document operationalizes nutritional counselling and IFAs objectives by providing guidelines for service providers. It provides nutrition interventions for women at different stages (pre-natal, antenatal, postpartum and continued care) and infants and children from conception to 5 years of age. Below we elaborate on the two most relevant policy implementation guidelines:

- Guideline 1: To strengthen maternal nutrition assessment and counselling, all pregnant women should have access to and should be knowledgeable about the need for an adequate and nutritious diet through nutritional training and counselling. They should be encouraged and supported on how to cope with the food-related problems during pregnancy, including morning sickness, constipation and heartburn.
- Guideline 2: Programme implementers should provide and promote intake of Iron and Folic Acid supplements (IFAs) through antenatal care services, and support other strategies to address maternal anaemia among women of reproductive age. Recommendations and key messages for this policy guideline include:
  - Encourage pregnant women to take 60 mg of Iron tablets daily for the duration of pregnancy irrespective of their haemoglobin levels to prevent anaemia.
  - Encourage mothers to continue to take 400 μg of folic acid daily around the time of conception to significantly reduce the incidence of neural tube defects Folic acid supplementation should be started in the first trimester of pregnancy to prevent birth defects.
  - Provide information on possible side effects and how to avoid them when taking IFA supplements.
  - Provide nutritional counselling practices that promote an iron-rich diet and absorption during pregnancy.

Despite that studies have proven efficacy of maternal nutrition education, counselling and supplementation in improving maternal nutrition [13], reducing infant and child mortality, improving physical and mental growth and development, and improving maternal health and pregnancy outcome in experimental settings [14–19], in practise the outcome in addressing maternal malnutrition and associated health indicators has been less successful than anticipated. Currently, Kenya is among the 10 countries that experience the most neonatal deaths globally and 42% of Kenyan pregnant women are estimated to be anaemic [2]. Besides, high levels of under-nutrition, particularly stunting, have persisted in Kenya for decades. The levels of wasting and stunting have remained unaltered for about 20 years at between 6 and 7% for wasting and 30 and 35% for stunting. Although Kenya has made significant strides in reducing neonatal, infant, child, and under-5 mortality, one in every 26 Kenyan children will die before reaching 1 year of age and one in every 19 will not survive to their fifth birthday [1, 2, 20]. This undoubtedly questions the implementation fidelity of guidelines on nutritional counselling and IFAs. There is therefore a pressing need to examine programme implementation processes in Kenya and to understand whether the guidelines are being implemented as intended.

The major reason for programme failure even among well-designed programmes is the failure to implement with fidelity. A meta-analysis of 500 studies from various fields showed that programmes with better implementation had mean effect sizes two to three times larger than those with poor implementation [13]. Therefore, this study aims to acquire insight into the degree to which the nutritional counselling and IFAs guidelines during pregnancy have been implemented as intended and which factors have constrained implementation fidelity. To this end we used a programme implementation fidelity framework and focused on Uasin Gishu County, Kenya, as a case study. Implementation research is one of the most important and at the same time most neglected aspects of programme evaluation research. Rather, outcome/impact evaluations have become the norm for most researchers, especially those studying maternal nutrition intervention programmes in Kenya [14–16]. The few studies that have focused on implementation fidelity of nutritional programmes, particularly in LMICs, mainly assessed participant responsiveness to the programme and left out other elements of fidelity [17–19, 21–23]. Obtaining a clear picture of how a programme was implemented is highly relevant because it not only allows programmers to more confidently link programmes to observed outcomes, but also provides important information on how programmes should be designed and implemented in future to produce positive results [24, 25].

## Methods

### Conceptual framework

**Fig. 1:**
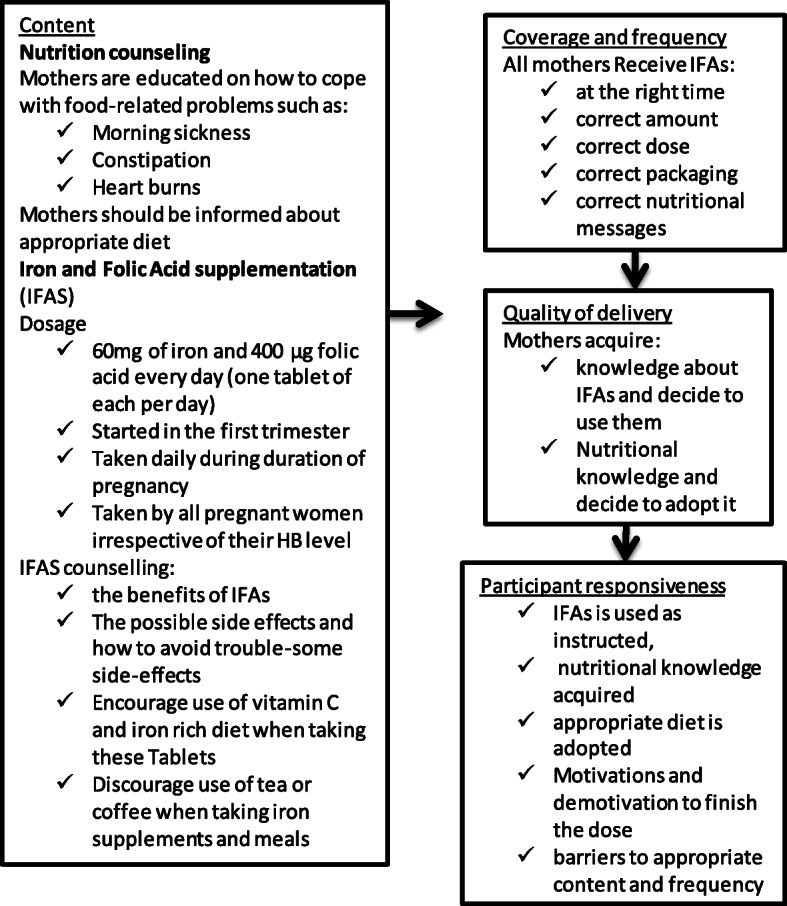
Programme theory of process pathways to maternal nutrition intervention in Kenya

We adopted ‘programme theory’ as the conceptual frame work to meet the main objective of this study. Programme theory as defined by Bickman is the construction of a plausible model of how a programme is supposed to work [26]. It involves the construction of a causal model linking programme inputs and activities to a chain of intended or observed outcomes and then using this model to guide the evaluation. There is no uniform way of developing such models because each is developed for a particular programme and does not represent the “off-the-shelf” use of a single established social science theory [26]. We used the program theory of MIYCN and next designed a programme implementation fidelity framework as presented in Fig. 1 to guide the evaluation. ‘Implementation fidelity’ (also termed programme integrity) is defined as the degree to which programmes are implemented as intended [25, 27].

To assess implementation fidelity, various main dimensions can be identified in literature, which Caroll et al. [28] grouped into two components:

1. *Adherence to intervention* – whether a programme service or intervention is being delivered as it was designed or written with central measures of fidelity: (a) content, (b) frequency, (c) duration, and (e) coverage;
2. *Moderating variables*, including (a) quality of delivery – how well the staff delivers a programme, and (b) participant responsiveness – how far participants respond to or are engaged by the intervention.

For this study, the conceptual interpretation of the programme theory and two core components of implementation fidelity – adherence and moderating variables – was guided by MIYCN implementation guidelines and messages as indicated in Fig. 1. This framework became a guiding tool in the design of research instruments, data analysis and interpretation of the results as further described in “data collection” section.

### Research design and study site

This institution-based descriptive cross-sectional survey used qualitative and quantitative methods of data collection. Researcher administered questionnaire was used to collect data from intervention recipients and a semi-structured interview guide was used to collect data from programme implementers. The study was conducted between March and June 2017 in rural Uasin Gishu County in the western part of Kenya (one of the 47 counties of Kenya). Uasin Gishu is predominantly inhabited by the Kalenjin who are the third largest ethnic community out of the 44 ethnic communities in the country [29]. The predominant settlement pattern in Uasin Gishu County is rural (64.1%) [29] and malnutrition in Kenya is highly prevalent in the rural areas [1, 2], hence the reason why this study was rural based. Data for this manuscript is part of a larger research project whose main objective was to investigate the social cultural context of nutrition in pregnancy and the utilization of nutrition intervention services in rural Uasin Gishu County [30].

### Study population and sampling

All major health facilities (sub-County hospitals) found in the rural areas of Uasin Gishu County – six in total – were selected for the study [31]. These health facilities normally serve as rural referral hospitals. Sub-County hospitals as a result receive the highest rural patient/client population from various locations in a given sub-County hence the reason why they were selected for the study. Respondents were recruited amongst the mothers seeking maternal care at the six sub-County hospitals of Uasin Gishu County.

Documenting women’s own experiences and perceptions on nutrition interventions and ANC services provided during their latest pregnancy was part of the main objectives of this study. As a result pregnant mothers who had at least one previous ANC services at a health facility and mothers who had delivered a baby within 1 month preceding the study were included in the study. To enhance cultural homogeneity, only Kalenjin mothers (the predominant cultural group in Uasin Gishu County) were included in the study. These selection criteria excluded: non-Kalenjin women, Kalenjin women seeking ANC for the first time or postnatal care past 1 month of delivery, as well as those unable or unwilling to participate.

Systematic sampling technique was used to select study participants whereby every second woman who met the inclusion criteria was recruited until the minimum desired sample size of 188 out of on average total of 795 women who were seen per month in these hospitals, was attained. Maternal care attendance registration database of 6 months preceding this study were used to determine the average number of women seeking maternal care per month in these health facilities.

### Data collection

#### Questionnaire

Researcher administered questionnaire, containing closed and open-ended questions, was used to collect quantitative and qualitative data, respectively, from the sampled respondents (intervention recipients). The conceptual framework guided the design of research instruments and data collection approaches. Prior to the study, a detailed literature search was conducted to understand programme theory guidelines and intervention strategies that are used in the implementation process. The framework was operationalized as follows.

- **Adherence**: Adherence was measured with the four components of Carroll et al’s model [28]: coverage, content, frequency, and duration.
  - To assess *coverage*, the number of respondents who ever turned up for MIYCN interventions at a health facility during their recent pregnancy was determined.
  - To evaluate whether interventions were delivered with the correct *content* as planned, the actual interventions received by these women based on MIYCN guidelines requirements was assessed, including the following indicators: general nutritional counselling, issuing iron and folic acid supplements (IFAs), and counselling on (a) how to deal with nutritional pregnancy related complications, and (b) uses of IFAs, including how to avoid troublesome side-effects of IFAs and how to increase IFAs absorption rate. The answers of respondents were validated by counterchecking information recorded in their appointment cards.
  - *Frequency* was calculated based on the total number of times women, who had recently delivered, had turned up for interventions at the health facility during their entire current gestation period and received the stated interventions. Self-reporting adherence assessment was adopted based on the total supplements issued in the prior ANC visits. Self-reporting adherence was validated by counting the remaining pills if any. The number of pills and the number of times issued was also validated by reading the recorded figures in the respondents’ clinic appointment cards. Attending at least four appointments and receiving a minimum 30 IFAs on each appointment as recommended by WHO was considered a cut-off for adequate adherence [12].
  - *Duration* of an intervention could not be determined as it was not included in the standard programme guidelines. Instead, the stage of pregnancy at which intervention was introduced was identified to be an important indicator and was adopted in this study.
- **Moderating variables:** Moderating variables comprised two components of Carroll et al’s model [28]: quality of delivery and participant responsiveness.
  - *Quality of delivery* - the quality by which nurses implement the elements of the programme – was assessed by determining the extent to which the recipients received the IFAs, as well as the knowledge of the intervention recipients on the received interventions (in this case knowledge on importance of IFAs, dosage prescribed for IFAs and the associated side effects).
  - To assess *participant responsiveness*, we used a number of indicators, including ANC attendance. For IFA supplementation, we asked if they finished the dose that was issued during the previous appointments and we validated this by physical count of the remaining pills. Open-ended questions were included in the questionnaire to establish the bottlenecks and strengths of the programme including: motivations and challenges for compliance to the supplements by programme recipients and implementers, sources of nutritional training and counselling other than the health facility.

The questionnaires were administered by the first author and trained research assistants to all mothers who met the inclusion criteria and consented to the study. The four research assistants were recruited with a background in social sciences, specifically degree in sociology. Before the actual data collection, 2 days theory and practical training regarding the objectives of the study and ways of administering the questionnaire were given to the data collectors by the first author. The prepared questionnaire was pre-tested prior to the actual data collection amongst ten pregnant women and eight post-natal mothers (*n* = 18) in one of the health facilities that was not included in the main survey. The questionnaire was edited and checked for completeness daily and before the data entry. All the research team was engaged in the pre-test and review of research instruments. The first part of the questionnaire largely contained close-ended questions. This part assessed the socioeconomic and demographic status of the respondents. It also assessed adherence, i.e. coverage, content, frequency and duration of the interventions, as well as anaemia status of the respondents. This information was retrieved from the respondent’s appointment card and the results were validated by the respondents. The second section of the questionnaire largely contained open-ended questions and it mainly assessed the quality of delivery and participant responsiveness.

Interviews were conducted in a room within the health facility, ensuring privacy. Detailed note taking and tape recording was done during the interviews which lasted for about 30–45 min. Respondents were encouraged to talk freely and clarifying questions were only asked when the women did not understand the questions or when the interviewers wanted to get insight story of the responses. The women were interviewed in Swahili or in their local language. The interviews were later translated and transcribed into English by one of the research assistants who understands both languages and validated by a professional translator.

#### Semi-structured interviews

Semi-structured interviews (SSIs) with programme implementers (nursing officers in charge of Maternal and Child Health (MCH) or whoever was on duty in the MCH section) were conducted to triangulate the research findings obtained from intervention recipients and document analysis. We specifically acquired information on programme theory and implementation as well as the challenges they face in the implementation of the MYICN guidelines. In total there were six programme implementers selected from each of the six sub-County hospitals where the study was conducted. The health workers were interviewed by the first author in the language of their preference (English or Swahili). The interviews were conducted in a quiet private room at their place of work to avoid distractions, ensure privacy and anonymity of the responses [32].

Information gathered from the SSIs was recorded, transcribed and further manually analysed to explore meanings and enrich the responses obtained from the interviews with mothers. Parts of these narratives have been presented in the results section.

### Data analysis

Collected statistical data were edited, coded, entered in Excel and exported to SPSS version 23. These were analysed to establish descriptive statistics such as frequency, percentage and mean which were used to describe studied variables. The first author performed the data coding and entry. Qualitative data were transcribed verbatim into Microsoft Word files and translated into English with each participant being identified with a pseudonym. Thereafter the transcripts together with field notes were transferred into MAXQDA 12.3.2 software for coding, analysis, and identification of major themes and sub-themes. Codes were based on main themes derived from the conceptual framework presented in Fig. 1 as the initial coding guide. The data were further coded based on recurring themes as identified in the transcripts of SSIs and the recurring issues raised by participants derived from the open-ended questions in the questionnaire. Thematic analysis was used to identify the most common recurring themes and issues [32, 33]. Several researchers with different backgrounds provided input in the analysis to increase its validity [34].

## Results

Presentation of the research findings follows the process pathways laid out in the programme theory (Fig. 1) and is divided into Carroll et al’s main dimensions of programme implementation fidelity evaluation [28]: For each step in the pathway we present quantitative and qualitative results in an integrated, complementary way. All data describing the social-demographic and health profile and implementation integrity of MYICN interventions were summarised using descriptive statistics and these are presented in Tables 1, 2 and 3.

### Social demographic characteristics of the questionnaire respondents

In total, 188 women participated in the survey of which 54% were pregnant and 46% were postnatal mothers. The age of the respondents ranged from 15 to 55 years with a mean age of 25.5 years old and the majority (85%) were married with multigravida (62%). Almost half (44%) of these women had primary education and three quarters (75%) worked in informal sectors either as farmers, housewives or small-scale entrepreneurs. 14% of the respondents were students either at secondary or post-secondary levels. Out of those women who were tested for Haemoglobin at the health facilities as recorded in their clinic appointment cards, 27% were anaemic (< 11 g/dl) and the mean HB status was 9.97 g/dl. These findings are presented in Table 1.

**Table 1:** Demographic and health profile of the respondents

Indicator	Characteristics of women	Distribution (n)	Percentage (%)	Mean
Selected demographic characteristics of the respondents (*n* = 188)	**Maternal status**			
	Pregnant	102	54	
	Recently delivered (RDM)	86	46	
	**Age of the respondent (years)**			
	15–19	15	8	
	20–24	79	42	
	25–29	59	31	
	30–34	22	12	
	35–39	10	16.5	
	≥40	3	2	
	Mean age			**25.5**
	**Educational level**			
	Primary	82	44	
	Secondary	75	40	
	Post-secondary	31	16	
	**Occupation**			
	Farmer	74	39	
	Housewife	24	13	
	Casual labourer	6	3	
	Business	44	23	
	Student	27	14	
	Salaried	13	7	
	**Marital status**			
	Never married	28	15	
	Married	160	85	
	**Gravida**			
	Primigravida	72	38	
	Multigravida	116	62	
Prevalence of anaemia among the participants (*n* = 188)	HB examination (*n* = 186)			
	Not tested for HB	26	14	
	Tested for HB	162	86	
	Mean haemoglobin (*n* = 162) concentration (g/dl^a^)			
	Severe anaemia (< 8)	6	4	
	Moderate anaemia (8–9.99)	16	10	
	Mild anaemia (10–10.99)	22	13	
	≥11	118	73	

^a^Grams per decilitre

### Implementation fidelity of MYICN

In line with the implementation fidelity framework, we first present components related to adherence to an intervention followed by moderating variables which covered quality of delivery and participant responsiveness.

#### Adherence

The first component in Carroll et al’s framework of implementation fidelity is adherence to the intervention; implementation coverage, content and frequency of counselling and IFAs with the exclusion of duration are presented in Table 2 and elaborated below.

**Table 2:** Content and frequency

	Characteristics of women	Distribution (n)	Percentage (%)
Interventions received: (*n* = 186)	**Supplements received**		
	Received Iron	138	74
	Received folic acids	88	47
	**Counselling received**		
	General nutrition information (*n* = 186)	52	28
	Information on iron (*n* = 138)	76	55
	Information on folic acid (*n* = 88)	38	43
Total folic acid supplement pills received for the entire period of pregnancy (based on RDM^a^) (n-86)	Never Received any	9	11
	< 15	3	3
	30	35	42
	60	22	26
	90	13	15
	> 90	3	3
Total iron pills received for the entire period of pregnancy (based on RDM^a^) (n-86)	Never received any	4	5
	< 15	13	15
	30	41	49
	60	12	14
	90	9	10
	150	6	7

^a^Recently Delivered Mothers

##### Coverage

Coverage for this study was based on the population covered by the intervention. As per the MYICN programme requirements, the intervention should cover all pregnant women across the country irrespective of their nutritional status. However, since the MYICN programme is implemented as an ANC integrated programme in government health facilities; it is only accessed by those women who seek routine Antenatal Care (ANC) services in these facilities. In this study, all respondents except two (RDM) had accessed ANC services at some point during their pregnancy period (which is not surprising since we recruited women at health facilities). However, as per findings of this study, only 10% of the respondents (intervention recipients) accessed ANC services during their first trimester of pregnancy, a majority attended after 5 or 6 months. Furthermore, not all women who had accessed health facilities for ANC were provided with the interventions recommended by the IFAs policy, thereby considerably reducing intervention coverage. Details are illustrated in the “content and frequency” section below.

##### Content and frequency

Regarding content we assessed the actual interventions received by these women based on MIYCN guidelines requirements: (1) general nutritional counselling, (2) issuing IFAs. With respect to frequency, we assessed the total number of times these women turned up for interventions at the health facility during their entire current gestation period and received the stated interventions.

The *general nutritional counselling* intervention received least attention by programme implementers. Only 28% of the respondents of the questionnaire reported to have received nutritional counselling on a general diet.

“I was not given any nutritional advice so I assumed my health condition was good. You know if blood is not enough or they notice that your health is not good they will advise you on what to eat.” (R31)

Long queues and staff shortages were the main reasons given by both programme implementers and intervention recipients for not providing or receiving nutritional counselling to all women:

“On that day the queue was too long. So I was not given any nutritional counselling.” (R18)

“We only provide nutritional counselling to those women who raise a nutritional concern or whose health is generally not good. Maybe their weight or HB is not adequate. Those who are HIV positive we refer them to AMPATH (Academic Model Providing Access to Healthcare) nutritionist for counselling. You cannot manage to provide individual counselling to everyone because they are too many and here you are providing so many services alone.” (Nursing officer attending to women at the MCH)

Women reported to have mainly acquired nutritional knowledge from other sources, including local women relations, school and own experience.

*IFAs provision* was clearly the main focus of MIYCN interventions. The IFAs interventions as a part of ANC were reported in at least 70% of women availing ANC (Table 2). The highest was iron supplementation (74%) and information on its usefulness (55%). The other elements of the interventions were reported by less than half of the questionnaire respondents.

Pregnant women are supposed to be encouraged to take IFAs daily during the duration of pregnancy irrespective of their haemoglobin levels (60 mg of iron and 400 μg folic acid per day) to prevent anaemia, receiving a minimum of 120 pills of iron and 120 pills of folic acid (4 months’ supply). However, only 3 and 17% of the respondents received more than 90 pills of folic acid and iron supplements respectively. This is a clear indication that women are not supplied with IFAs in every ANC appointment as recommended by MIYCN.

Stock shortage was the major challenge that affected frequency of supplementation as mentioned by a programme implementer:

“Sometimes like now we have very few pills in the drug store and I don’t foresee the possibility of receiving the stock any time soon. In such circumstance, we prioritise those women whose HB is low. Others we give them half of the required dose at least to ensure equitable distribution.” (nursing officer in charge of MCH)

This was confirmed by one of the intervention recipients:

“They checked my blood (HB) and they found it was sufficient so I wasn’t given any drugs (supplements).” (R24)

Other facilities did not have any stock at all:

“We have run short of stock for the past three months. It is more than four months now ever since we placed the order. In this case we prescribe supplements to the women and advise them to buy from the drug stores in the market………..With the County government, procurement process takes too long.” (Nursing officer in charge of a health facility)

#### Moderating factors

The moderating factors assessed in this study relate to quality of delivery and participant responsiveness to the intervention. Various indicators were assessed as presented in Table 3.

**Table 3:** Participant responsiveness and quality of delivery

	Characteristics of women	Distribution (n)	Percentage (%)	Mean
Coverage of interventions	Did not access any ANC	2	1	
	Accessed health ANC	186	99	
Initial access to interventions	**Gestational age at first ANC visit (weeks) (*n* = 186)**			
	< 13 weeks	18	10	
	13–19.9	27	14	
	20–26.9	95	51	
	≥27	46	25	
	Average			23.4 weeks
Frequency of access to ANC services (based on RDM) Number of times (*n* = 86)	**Number of times (*n*> = 86)**			
	1	4	5	
	2	14	16	
	3	30	35	
	4	22	26	
	≥5	16	19	
Adherence to the supplements issued in prior ANC visits	Finished iron supplements	94	68	
	Finished folic acid	56	64	
Reasons for non-adherence to supplements	**Iron (*n* = 44)**			
	Side effects	25	57	
	Delivered before finishing	8	18	
	Drugs were too many	6	14	
	Haemoglobin was okay	3	7	
	Confusing dose	1	2	
	Was using traditional herbs	1	2	
	**Folic Acid (*n*** **= 32)**			
	Side effects	19	59	
	Delivered before finishing	5	16	
	Haemoglobin was good	3	10	
	Drugs were too many	2	6	
	Made her sleep a lot	1	3	
	Forgetful	1	3	
	She stopped vomiting	1	3	
Motivations for adherence to supplements	**Iron (*n* = 94)**			
	Its usefulness	43	46	
	Obeyed doctor’s instructions	30	32	
	Was sick	12	13	
	Did not experience side effects	9	9	
	**Folic Acid (*n*** **= 56)**			
	Its usefulness	24	43	
	Obeyed doctor’s instructions	20	36	
	Was sick	8	14	
	Did not experience side effects	4	7	
Knowledge on Iron supplementation (uses) (*n* = 138)	Increases amount of blood of the mother	110	80	
	I don’t know	19	13	
	Eliminates the urge of eating soil	5	4	
	Development of the baby	4	3	
Knowledge on Folic Acid supplementation (uses) (*n* = 88)	I don’t know	36	40	
	Development of the baby	19	22	
	Bone formation and strengthening	6	7	
	Increases mother’s amount of blood	5	6	
	Improves mother’s appetite	4	5	
	Prevents the baby from developing deformities	4	5	
	Multivitamins	3	3	
	Gives energy to mother	3	3	
	Anti-malaria	2	2	
	Immunity booster	2	2	
	Reduces heartburns	2	2	
	Spine formation of the baby	2	2	
	Prevents vomiting and nausea feelings	1	1	
Sources of general nutrition knowledge (*n* = 188)	own knowledge	69	37	
	Other women	31	16.5	
	Health officer	30	16	
	Learned in school	21	11	
	Experiences from previous pregnancy	13	7	
	Self and hospital	11	6	
	School and hospital	7	4	
	Other women and hospital	5	2	
	Radio	1	0.5	
Dosage prescribed Ferrous Iron (*n* = 138)	one pill daily	83	60	
	one pill three times a day	28	20	
	one pill twice a day	17	12	
	two pills once a day	6	4	
	two pills three times a day	4	3	
Dosage prescription Folic acid (*n* = 88)	one pill daily	57	65	
	one pill twice a day	15	17	
	one pill three times a day	12	14	
	two pills three times a day	4	4	

##### Quality of delivery

For those who were supplied with supplements, the dosage prescribed to them varied considerably. Correct dosage of one supplement daily was reported by 83 and 57% of the respondents for Iron and FA respectively as indicated in Table 3. One of the programme implementers clarified the differences in dosage in iron supplementation:

“…. If the tested HB status reads below 10 g/dl, we give a prescription of one pill three times a day, after one month we re- test HB, if it has improved, we reduce the dose to one pill daily for the remaining months until birth” (Nursing officer at a health facility)

On the other hand, one programme implementer felt that too many drugs are not healthy to a pregnancy:

“You know these are chemicals: too much chemicals are not healthy to human bodies especially when pregnant. When a woman’s HB is more than 13g/dl I don’t see the need of pumping her with chemicals so in that case I don’t issue the supplements.” (Nursing officer working in MCH at a hospital)

Shortage of pills not only affected adherence but also quality of delivery by the health providers.

##### Participant responsiveness to the intervention

We investigated the degree to which pregnant women embraced the interventions and this was measured by access to interventions, adherence to the intervention instructions and their understanding of the interventions.

- *Access to interventions:* According to the MYICN guidelines, interventions should be initiated around the time of conception to increase their efficacy. Each woman is also expected to make at least four ANC appointments during the entire period of pregnancy. In this study, as indicated in Table 3 it was established that out of 186 women who availed for ANC services only 10% initiated contact with the ANC within the first trimester and only 45% made four or more appointments during the entire period of pregnancy (based on the recently delivered women).
- *Adherence to the intervention:* According to the self-reported adherence, 68 and 64% of the respondents finished the pills issued for iron and folic acid supplements respectively during the previous appointments. Usefulness of the supplements (46 and 43% respectively) and obedience to the doctor’s instructions (36 and 32% respectively) were the major motivating factors to complete the dose.
  “I know during birth a woman loses a lot of blood. I had to finish taking them because I wanted to be on a safer side by having enough blood back-up.” (R9)
  “During my last birth, I underwent an operation and lost a lot of blood. I really had to finish the pills to ensure I have enough blood back-up for the next birth.” (R30)
  “Actually, I don’t know its usefulness. Because it is issued by a learned informed doctor, I believed it must have some importance to a pregnancy so I decided to finish taking them.” (R19)

On the other hand, anticipated and experienced side effects were the major reason reported by more than 50% of the respondents for non-adherence.

“It leaves a bad lasting annoying smell in the throat when you take them. I just don’t like taking them.” (R7)

“My HB status was good so I did not bother taking them. You know how they are bad.” (R18)

Others respondents gave other reasons, such as use of other (traditional) drugs:

“I was already taking other drugs that I was given to prevent miscarriage. So I felt that the drugs have become too many and I decided to stop taking the supplements I first finish the previous drugs which I felt were more important.” (R1)

“My husband had bought for me herbal medicine. I decided not to mix taking both. I decided to finish the herbs first then I continue with the hospital pills.” (R26)

Or the perceived high number of pills:

“I did not finish them; they were too many. I gave birth but still many were left over.” (R20)

Another reason for poor adherence resulted from confusion about the dosage variation, which was not explained by the health worker. One pregnant respondent reported that:

“The first time when I came for ANC services the nurse gave me 30 pills and told me to take one pill per day. The second time I got a different nurse. She gave me so many pills and advised me to take one three times a day. I now got confused. I decided to follow the previous dose prescription. That is why you can see I still have so many unfinished pills.” (R2)

Other motivational and demotivating reasons for adherence to the supplements are presented in Table 3.

- *Understanding of the intervention.* A large majority of the respondents (80%) had correct knowledge on the importance of consumption of iron supplements, unlike folic acid supplementation where 40% of the respondents did not understand the importance of taking it. Mixed responses from intervention recipients on the importance of taking folic acid emerged as presented in Table 3.
  “I know it helps in the development of the baby but I don’t know how.” (R66)
  “It helps to relief heartburns. When I was taking them, it relieved my heartburns so I continued taking them.” (R171)
  “It helps one to sleep. I used to sleep a lot and that is why I stopped taking them.” (R146)
  “It reduces that urge of wanting to eat soil (pica).” (R170)
  “It prevents malaria.” (R151)

On the other hand, multiple sources of nutritional knowledge were reported by the respondents and this is likely to compete with hospital knowledge and may affect adherence. Only 16% of the interviewed respondents reported to rely on the knowledge acquired at the hospital. The highest number of the respondents rely on own-knowledge (37%) and the knowledge acquired either from other women relations (16%) or from school (11%) and others combined knowledge acquired from the several sources.

## Discussion

The aim of the study was to assess the implementation fidelity of the on-going nutritional counselling and IFAs intervention policy and the constraints encountered.

Although the overall coverage level among the respondents amounted to 99%, which is slightly higher than the 96% of Kenya overall [1], the late initial access to ANC impacts negatively on implementation fidelity. Guidelines recommend interventions to be initiated around the time of conception or in the first trimester to enhance effectiveness of nutritional counselling and supplement usage. However, the findings indicate a large percentage of women (90%) did not seek ANC during their first trimester. This percentage is higher than the 80.2% in Kenya overall [1]. Late booking of ANC is also a common trend in other parts of Africa [30, 35–37] for various reasons and this strongly affects frequency fidelity and effectiveness of interventions. Khadim (2007) established a correlation between early registration to ANC services and iron supplement consumption among pregnant women in Senegal [21].

Implementation fidelity was also evaluated through the content of services received and the kind of information mothers were given during their visit. The women we interviewed reported to have received all the components of the programme including nutritional counselling and supplementation. However, partial provision was reported in all the intervention components. Only iron supplementation was relatively high with 74% of the respondents being given iron supplements and 80% understood its usefulness. This was a higher proportion compared to 47% who received folic acid, of which almost half (43%) did not have any idea about its usefulness while most others reported a wide variety of misconceptions. Lack of knowledge on folic acid supplements is common among pregnant women in LMICs for example in Kenya and Croatia where 59.1 and 48% of the respondents respectively did not know what FA is [28, 38].

Frequency integrity was low, only 18 and 15% of the respondents received 90 or more iron and folic acid pills respectively during their entire pregnancy period. However, based on a standard dose of 60 mg iron and 400 μg folic acid daily for 6 months, each woman should receive a minimum of 180 pills in the entire pregnancy period. Stock shortage was the main reported reason for low frequency of supply of nutritional supplementation, a similar finding to a study in Nyeri County in Kenya [19].

Of those respondents that did receive nutritional supplements, not more than 68% completed taking the supplements that were issued to them in the previous ANC appointments. Side effects of the pills were the main reason reported for low compliance to IFAs in this study and in other studies in African counties [18, 23, 39]. Despite IFAS side effects being a leading contributor to poor compliance, less than half of the respondents reported having received counselling related to IFAs. A similar finding was established in Nyeri County in Kenya where 58% received counselling information about IFAs [19]. Counselling and knowledge on IFAs has been established in literature to have a significant association with adherence [17, 21, 38, 40–42]. Similarly knowledge on usefulness of IFAs and health provider’s strict instructions were established to be the greatest motivation to adherence by almost half of the compliant respondents. It is therefore critical that clients are properly counselled on the possible side effects when IFAS tablets are issued as well as their management and the importance of adherence to dosage.

The fact that IFAs does not cover all pregnant women and that compliance to IFAs is relatively weak points to the compelling and vital role of nutritional counselling to promote locally available, affordable micro-nutrient rich food sources. However, only 28% of our study population reported receiving counselling on diet during the previous ANC appointments. It was also established that nutritional counselling was only provided to those women whose health status was generally not good. This could limit the gains expected out of these sessions on nutritional knowledge that is critical in early stages of pregnancy to achieve greater impact on health. Only 16% of the respondents rely on nutritional knowledge acquired at the hospital. Most women (37%) rely on own knowledge regarding appropriate nutrition or knowledge acquired from other women and/or learned in school. This knowledge acquired from multiple alternative sources might conflict with that provided by the health providers and this might affect compliance. A similar finding was established among the Ghanaian pregnant women whose knowledge about food was drawn from multiple sources, some of which were in line with hospital knowledge while others conflicted [43]. Knowledge acquired from local women in most cases tends to be restricted to low-cost dietary sources of micronutrients which are readily available in their environment due to cultural nutritional taboos [44, 45].

The main reason reported by programme implementers for not providing nutritional counselling to all intervention recipients was a long queue; a sure sign of a shortage of health workers. Considering that ANC services are provided free of charge in government health facilities, overcrowding is highly likely to be experienced especially in the rural areas. Studies from low resource settings have also established the severe shortage of health workers hinder the capacity of health systems to deliver the required health services [46].

There were other minor challenges to implementation fidelity that were reported which included, forgetfulness, being supplied with many pills, prescription of contradicting dosage, others gave birth before finishing the dose and others discontinued taking pills when their health condition improved.

## Conclusion

Implementation fidelity of the nutritional policy guidelines of the MYICN programme for pregnant women in Kenya is generally weak. This is mainly experienced in the content and frequency elements. Late initial booking to ANC, stock shortage, staff shortage (and resulting long queues), side effects of drugs and issuing of many pills at ago were established to be the main factors affecting implementation fidelity.

## Recommendations

One of the factors affecting adherence to interventions in this study and other studies in Africa is the late initial booking to ANC services. Thus there is need to investigate and address the factors attributing to the late booking of ANC services.

Drug shortage due to complicated procurement and supply procedures was also established to be the major factor affecting adherence. There is therefore need to shorten the procurement processes in order to ensure availability of the supplements at all times. In additions it is important to strengthen the nutritional counselling component on general diet. This will promote the consumption of micronutrient-rich food sources available in the local environment and may substitute the shortage of nutritional supplements that is commonly experienced in the health facilities in the study area and other regions of the country.

Most women discontinue IFAS whenever they experienced side effects. Ability to manage IFAS side effects will result in higher compliance. It is therefore critical that women are properly counselled on the possible side effects of IFAS pills and how to manage them.

Another reason cited for poor compliance to IFAs was, supply of many pills. This is probably because iron and folic acid pills are issued as two separate tablets. Combination of iron and folic acid into one tablet should be done to reduce pill burden thus increase compliance.

Most women reported that they acquired nutritional knowledge from multiple sources; mainly from their own experience, school or other women. There is therefore a need to carryout investigations on the content and quality of the nutritional knowledge acquired from these sources, and to what extent this supports or conflicts with information based on the nutrition guidelines. In addition, would be interesting to investigate the advantages of targeting these women advisors in the community and empowering them to provide appropriate nutritional counselling to pregnant women, given their role is a trusted source of nutritional information. Knowledge provided by women advisors will not only be more trusted by pregnant women, but will also reach out to women within their first trimester of pregnancy as recommended by MIYCN programme.

### Limitations and strengths of the study

Data collected for this study was based on self-reported information collected directly from intervention participants using a checklist of the components of intervention protocols. Data collected based on self-reports provides important clinical information regarding the viability of the intervention during dissemination and is useful in designing future versions of the programme [47]. However, data based on self-reported measures may have potential limitations related to accuracy. Distortions in data may occur due to poor recollection by participants. In addition, participants may have biased feelings towards the implementer and give socially desired answers. This was countered by validating the responses with the information in the clinic appointment cards and seeking clarifications from programme implementers on issues that were not clear.

On the other hand, data for this study only assessed adherence to the implementation of the core components of the programme. It is also important for future studies to consider assessing the competences with which the practitioners deliver these core components to the recipients and more contextual factors that could affect the quality of implementation.

The results of this study were based on the findings from rural Uasin Gishu County (the case selected for this study). However, the findings can be generalized to other rural areas in Kenya and other LMICs. Rural areas in most LMICs, are faced with common institutional and infrastructural challenges such as: staff shortage, shortage of drugs, late and infrequent access to ANC for interventions, reliance on multiple sources for nutritional knowledge, hence the findings of this study can be generalized to such settings.

## Data Availability

Freely available data: data in tables and summary form in results and anonymised quotations presented in the papers. Access restrictions will apply to: Interview transcripts, Interview data, field notes and audio data. This is because, these data contain sensitive information of the respondents and may also contain identifiable information, that may be a threat to confidentiality and breach of data sharing agreements included in the consent forms used in this study. As a result, additional approvals will be required from the ethical review committees to re-use data, and in keeping in line with informed consent agreements. Applications can be submitted to the Kenya National Commission for Science Technology and Innovation (customercare@nacosti.go.ke / info@nacosti.go.ke).

## Abbreviations

MNCH: Maternal newborn and child health
IUGR: Intrauterine growth retardation
LBW: Low birth weight
LMICs: Low and middle income countries
IGR: Intrauterine growth retardation
WHO: World Health Organization
FANC: Focused antenatal care
MCH/FP: Maternal child health/family planning
MIYCN: Maternal infant and young child nutrition
IMAM: Integrated management of acute malnutrition
ANC: Antenatal care
IFAs: Iron and folic acid supplementation
HB: Haemoglobin
KDHS: Kenya Demographic and Health Survey
ICU: Intensive care unit
PNC: Postnatal care
SSI: Semi-structured interview
MCH: Maternal and Child Health
